# Screening breast cancer patients for Norwegian *ATM* mutations

**DOI:** 10.1054/bjoc.2000.1519

**Published:** 2000-12

**Authors:** K Laake, P Vu, T I Andersen, B Erikstein, R Kåresen, P E Lønning, E Skovlund, A L Børresen-Dale

**Affiliations:** Department of Genetics, The Norwegian Radium Hospital, Montebello, Oslo, 0310; Department of Oncology, Ullevål University Hospital, Oslo, 0407; Department of Oncology, The Norwegian Radium Hospital; Department of Surgery, Ullevål University Hospital; Department of Oncology, Haukeland Hospital, POB 1, Bergen, 5021; Clinical Research Office, The Norwegian Radium Hospital, Norway

**Keywords:** ataxia telangiectasia, breast neoplasms, heterozygote detection, polymerase chain reaction, breast cancer risk, Norwegian founder mutation

## Abstract

483 Norwegian breast cancer patients were screened for six different ataxia telangiectasia mutated (*ATM*) mutations previously found to account for 83% of the disease alleles in Norwegian ataxia telangiectasia (AT) patients. Only one carrier was found. These results provide no evidence in favour of an excess risk of breast cancer associated with heterozygosity for classical AT mutations, but remain consistent with a maximum 2.4-fold increased risk. © 2000 Cancer Research Campaign http://www.bjcancer.com


*British Journal of Cancer* (2000) **83**(12), 1650–1653
© 2000 Cancer Research Campaign
doi: 10.1054/ bjoc.2000.1519, available online at http://www.idealibrary.com on IDEAL®

http://www.bjcancer.com

### Screening breast cancer patients for Norwegian *ATM* mutations

**K Laake[1], P Vu[1], TI Andersen[2], B Erikstein[3], R Kåresen[4], PE Lønning[5], E Skovlund[6] and AL Børresen-Dale[1]**

[1]Department of Genetics, The Norwegian Radium Hospital, Montebello, 0310 Oslo; [2]Department of Oncology, Ullevål University Hospital, 0407 Oslo; [3]Department of Oncology, The Norwegian Radium Hospital; [4]Department of Surgery, Ullevål University Hospital; [5]Department of Oncology, Haukeland Hospital, POB 1, 5021 Bergen; [6]Clinical Research Office, The Norwegian Radium Hospital; Norway

**Summary** 483 Norwegian breast cancer patients were screened for six different ataxia telangiectasia mutated (*ATM*) mutations previously found to account for 83% of the disease alleles in Norwegian ataxia telangiectasia (AT) patients. Only one carrier was found. These results provide no evidence in favour of an excess risk of breast cancer associated with heterozygosity for classical AT mutations, but remain consistent with a maximum 2.4-fold increased risk. © 2000 Cancer Research Campaign http://www.bjcancer.com

**Keywords**: ataxia telangiectasia; breast neoplasms; heterozygote detection; polymerase chain reaction; breast cancer risk; Norwegian founder mutation

Ataxia telangiectasia (AT) is an autosomal recessive disorder caused by mutations in the *ATM* gene (Savitsky et al, 1995). It is characterized by ataxia, telangiectasias, immunodeficiency, chromosomal instability, radiation sensitivity, and an increased incidence of malignancies, primarily of lymphoid origin (for review *see* Boder, 1985; Regueiro et al, 2000). AT is diagnosed in approximately 1:40 000–300 000 live births in various ethnic groups, whereas about 0.35–1% of the general population is heterozygous (Sedgwick and Boder, 1991; Swift et al, 1991; Taylor et al, 1994).

Heterozygotes are clinically asymptomatic, but a number of epidemiological studies in AT families have noted a significant increase in cancer incidence, in breast cancer particularly (Easton, 1994; Athma et al, 1996; Inskip et al, 1999; Janin et al, 1999; Olsen et al, 2000). The relative risk was in the range 1.5–9, depending on population, age and family relation. Interestingly, cancer deaths do not appear to be significantly increased. Few of these studies have analysed carrier status and the number of observed breast cancer cases is relatively low, resulting in low statistical power.

A broad spectrum of breast cancer patients have been screened for mutations in the *ATM* gene: unselected series, patients with early-onset and late-onset disease, patients in cancer families, patients showing adverse reactions to radiation therapy, a cohort with frequent occurrence of bilateral disease, and Hodgkin's patients who developed breast cancer after radiation treatment. Only two of these studies found proportions of truncating mutations above the population estimate. Vorechovsky et al (1996*a*) screened 88 breast cancer patients in cancer families. Three index cases carried truncating mutations, but the mutations did not consistently segregate with the disease in the families. Broeks et al (2000) found seven truncating mutations in 82 breast cancer patients characterized by frequent bilateral occurrence, early age-of-onset, and long-term survival. Three of these patients carried the same intronic alteration, IVS10-6 G/T, the function of which is uncertain. Several studies did not find an excess of classical *ATM* mutations in breast cancer patients (Vorechovsky et al, 1996*b*; Appleby et al, 1997; Fitzgerald et al, 1997; Chen et al, 1998; Shayeghi et al, 1998; Bebb et al, 1999; Izatt et al, 1999; Nichols et al, 1999; Oppitz et al, 1999). The statistical power is generally low in these studies due to small numbers of *ATM* mutation carriers (Bishop and Hopper, 1997) and most studies have screened for classical truncating mutations only. There is a possibility that the mutation spectrum in breast cancer patients is different from the spectrum in AT patients. Gatti et al (1999) hypothesize that missense mutations result in breast cancer in heterozygotes, but not in classical AT in homozyogtes.

The aim of the present study was to screen a large number of Norwegian breast cancer patients for six unique *ATM* mutations found in Norwegian AT patients. The six mutations constituted 83% of the disease alleles in the Norwegian AT patients (Laake et al, 1998, 2000). One single mutation constitutes 57% of the disease alleles. Three others were recurrent in the Nordic countries (Table 1). The ascertainment of the AT patients was population-based in the years 1975–1994 (Olsen et al, 2000), and we assume that the majority of the *ATM* disease-causing alleles in our population are represented by these six mutations. A simple multiplex PCR analysis was developed to screen for these mutations with a high throughput.

### MATERIALS AND METHODS

The breast cancer patients belonged to three different series collected at different hospitals. The Norwegian Radium Hospital, Oslo, 295 cases (Andersen et al, 1994); Ullevål University Hospital, Oslo, 136 cases (Bukholm et al, 1997); and Haukeland

*Received 2 December 1999*
*Revised 21 August 2000*
*Accepted 6 September 2000*

*Correspondence to:* AL Børresen-Dale; E-mail: alb@radium.uio.no

**Table 1** Description of the Norwegian *ATM* mutations analysed

| Genomic sequence alteration | Exon | mRNA alteration | Allele frequency among AT families: Norwegian | Allele frequency among AT families: Nordic |
|---|---|---|---|---|
| 3245–3247 delATC insTGAT | 24 | Frame shift at codon 1082 | 16/28 | 16/82 |
| 4632–4637 delCTTA | 33 | Nonsense, Y1544X | 1/28 | 3/82 |
| 7875–7876 TG/GC | 55 | Substitutions, DA2625–2627EP | 1/28 | 1/82 |
| 8264–8268 delATAAG | 58 | Skipping of exon 58 (39aa) | 2/28 | 2/82 |
| 8432 delA | 60 | Frameshift at codon 2811 | 3/28 | 3/82 |
| 8978–8981 delGAAA insAT | 64 | Frameshift at codon 2993 | 1/28 | 1/82 |
| Total frequencies | | | 24/28 | 26/82 |

Hospital, Bergen, 52 cases (Aas et al, 1996). The median age at diagnosis was 59 years, range 27–90 years.

The six mutations screened for are described in Table 1 (Laake et al, 1998, 2000). Genotyping was performed in microtitre plates by a multiplex PCR in a 25 μl reaction volume containing 50–200 ng leukocyte DNA, 1 × Perkin Elmer buffer I, 4 mM $MgCl_2$, 0.15 mM of each of the dNTPs, 0.04 U AmpliTaq, 0.2 μM of each primer, except for the primers ATEX60F and ATEX60R of which 0.6 μM was used. Primer sequences are shown in Table 2. The PCR programme was: 94˚C for 2 min; 30 cycles of 30 s at 94˚C, 45 s at 56˚C and 45 s at 72˚C; followed by 2 min at 72˚C, 1 min at 94˚C and 60 min at 65˚C to allow heteroduplex formation. The PCR products were electrophoresed in a 7.5% polyacrylamide gel (1:37.5 bis content) at 150 V for 50 min in 1 × TAE buffer. The gel was stained with ethidium bromide and photographed under UV light. All cases showing aberrantly migrating bands were sequenced.

Exact 95% confidence intervals for the mutation frequency were estimated by StatXact (binomial distribution). Power estimations were performed by normal approximation to the binomial distribution (nQuery).

**Table 2** Primer sequences

| | |
|---|---|
| EX23F2 | GGC ATC TAA CAA AGG AGA GG |
| EX24R2 | TGT AAG ACA TTC TAC TGC CAT C |
| EX33F | CAC AGA AAC TAA AAG CTG GGT A |
| EX33R | TGC CTG GCC TAC GTA TAT |
| EX55F | TGT TGG GTA GTT CCT TAT GT |
| EX55R | CAA GGG CAG TTT TAG TAA C |
| EX58F | ATG AAA GAA TGG CAG TAG GT |
| EX58R | CCT CCC AAA GCA TTA TGA |
| EX64F | CTC AAG GAA ACA TGA AGT GTG |
| EX65R | GCA GAG ATG TTC CTT AAG ACC |
| EX60F | TGC CCC TAT ATC TGT CAT AT |
| EX60 | CTC AAT CTA CTA TAT GTA CAA G |

**Table 3** The power to detect a hypothetical relative risk of breast cancer

| Hypothetical relative risk | Power (%) in all ages[a] | Power (%) in ages < 55[b] |
|---|---|---|
| 2 | 38 | 21 |
| 3 | 74 | 44 |
| 4 | 91 | 62 |
| 4.6 | 95 | 70 |
| 5 | 97 | 75 |
| 6 | 99 | 83 |
| 7 | 99 | 89 |
| 8 | 99 | 93 |
| 9 | 99 | 95 |

[a]Number investigated is 483; [b]Number investigated is 150.

### RESULTS

A simple multiplex PCR was used to screen 483 breast cancer patients for presence of six *ATM* mutations seen in homozygous AT patients. The migration patterns of the different mutants are shown in Figure 1. Only one breast cancer patient (ULLB-120) was found to carry an *ATM* mutation, the Norwegian founder mutation (3245–3247 delATC insTGAT). The patient was first diagnosed with a lobular breast carcinoma (T2, N0, M0) at the age of 44. Four years later she developed a tubular carcinoma (T1, N0, M0) in the contralateral breast. The patient was alive and disease-free at age 52. No family history of breast cancer in first-degree relatives was recorded at time of diagnosis. Her first carcinoma did not exhibit LOH at any of seven microsatellite markers within and surrounding the *ATM* gene (Laake et al, 1997).

The frequency of classical *ATM* mutations in the patients analysed was 1/483, i.e. 0.2%; 95% CI = 0.01–1.2. According to the upper limit of this estimate of 1.2%, and the 0.5% estimated population frequency for *ATM* heterozygosity in the Nordic population (Olsen et al, 2000), the present data are consistent with a maximum of 2.4-fold increased lifetime risk of breast cancer. The present study had 95% power to detect a 4.6-fold elevated lifetime risk according to the number of cases analysed, estimated by one sample two-sided chi-square test at a 5% significance level (Table 3).

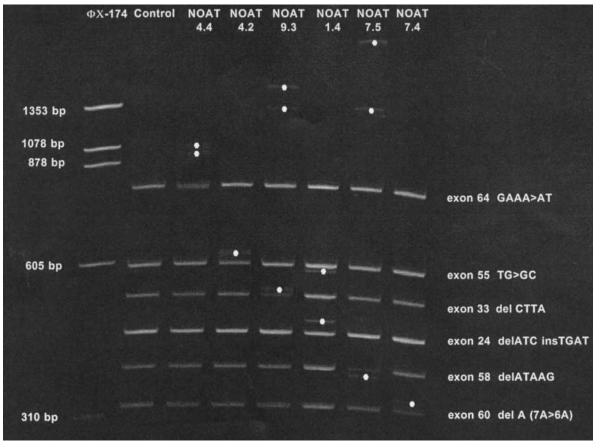

**Figure 1** A single multiplex PCR can detect any of the six Norwegian *ATM* mutations in one reaction. The mutant alleles are detected as a distinct heteroduplex pattern when the PCR product is electrophoresed in a 7.5% acrylamide gel. The heteroduplexes are marked with dots, and the mutant sequences are written to the left. Each of the samples loaded was carrying one of these six mutations.

© 2000 Cancer Research Campaign

### DISCUSSION

In the epidemiological study of cancer risk in Nordic AT relatives, the highest standard incidence ratio (SIR) of breast cancer, 8.5 (95% CI = 2.7–20), was found among mothers of AT children, aged below 55. The present study had more than 95% power to detect the point estimate of 8.5, but only 38% power to detect the lower 95% confidence limit of 2.7 (Table 3). Concerning lifetime risk of breast cancer, mothers again showed the highest SIR, 7.1 (95% CI = 2.3–17). The present study had more than 95% power to detect a 7.1-fold increased lifetime risk of breast cancer, but only 51% power to detect the lower 95% confidence limit (2.3) of the estimated SIR.

There is a possibility that *ATM* mutations are carried by a subgroup of breast cancer patients with distinct characteristics. Most of the epidemiological studies of AT relatives report an increased risk of breast cancer at younger ages, below 45–55 years (Inskip et al, 1999; Janin et al, 1999; Olsen et al, 2000), contrary from Athma et al (1996). In the present study, only 150 of the patients analysed were below the age of 55 at time of diagnosis, which may explain the low frequency of mutations seen. Among the patients analysed in the present study, about 25% of the cases were diagnosed with advanced breast cancer (stages III and IV). However, there is no evidence that breast cancer patients carrying *ATM* mutations are diagnosed with advanced cancer. A subgroup of patients found to carry *ATM* mutations is those with radiation-induced bilateral breast cancer at young age with good prognosis (Broeks et al, 2000). However, no excess of truncating *ATM* mutations were found in three small cohorts of breast cancer patients showing tissue radiation side-effects (Appleby et al, 1997; Shayeghi et al, 1998; Oppitz et al, 1999).

The studied mutations have been associated with breast cancer in AT relatives. Eight cases were identified among the Norwegian relatives (Olsen et al, 2000). One of them had the Norwegian founder mutation and the other the deletion in exon 33 (unpublished results). Carrier status of the other cases are still unknown. Vorechovsky et al (1996*a*) also found one woman with breast cancer carrying the Norwegian founder mutation. No missense mutations were screened for in the present study. There are, however, several reports on germline amino acid substitutions in breast cancer patients with frequencies ranging from 7–41% (Vorechovsky et al, 1996*a*, 1996*b*; Appleby et al, 1997; Larson et al, 1998; Shayeghi et al, 1998; Izatt et al, 1999). Gatti et al (1999) hypothesize that both heterozygotes and homozygotes for the two types of *ATM* mutations, the truncating ($ATM^{trunc}$), resulting in no protein or truncated protein, and the missense ($ATM^{mis}$) resulting in reduced amounts of defective protein, may give different phenotypes. The phenotype of $ATM^{trunc/trunc}$ mutations is the AT syndrome, while the phenotype of $ATM^{trunc/mis}$ is more unclear but with elevated cancer risk. Carriers of $ATM^{trunc/wt}$ and $ATM^{mis/wt}$ mutations may both be at risk for breast cancer, but the frequency of the $\mathrm{ATM}^{mis/wt}$ will be much higher in the population than the $\mathrm{ATM}^{trunc/wt}$ carrier.

It is not evident whether *ATM* acts as a tumour suppressor gene or not. None of the tumours showing LOH in the *ATM* region in a previous study (Laake et al, 1997) carried any of the six analysed mutations. This is in agreement with Vorechovsky et al (1996*b*). Recently, Izatt et al (1999) reported loss of the wild type allele in five breast cancer patients carrying different missense substitutions in the ATM protein, suggesting that LOH coincides with *ATM* missense mutations in breast cancer development.

The effect of the different missense substitutions on the function of the ATM protein is unknown. Some of these substitutions most likely induce minimal changes (polymorphisms), whereas others abrogate protein function. Functional studies, extended knowledge of the ATM protein structure and function, in addition to case control studies, will elucidate whether carriers of these variants are at risk of developing breast cancer.

### ACKNOWLEDGEMENT

Kirsten Laake is a research fellow of the Norwegian Research Council. We acknowledge financial support from Sigurd K. Thoresen's legacy.

### REFERENCES

Aas T, Børresen AL, Geisler S, Smith-Sørensen B, Johnsen H, Varhaug JE, Akslen LA and Lønning PE (1996) Specific P53 mutations are associated with de novo resistance to doxorubicin in breast cancer patients. *Nat Med* **2**: 811–814

Andersen TI, Heimdal KR, Skrede M, Tveit K, Berg K and Børresen AL (1994) Oestrogen receptor (ESR) polymorphisms and breast cancer susceptibility. *Hum Genet* **94**: 665–670

Appleby JM, Barber JB, Levine E, Varley JM, Taylor AM, Stankovic T, Heighway J, Warren C and Scott D (1997) Absence of mutations in the ATM gene in breast cancer patients with severe responses to radiotherapy. *Br J Cancer* **76**: 1546–1549

Athma P, Rappaport R and Swift M (1996) Molecular genotyping shows that ataxia-telangiectasia heterozygotes are predisposed to breast cancer. *Cancer Genet Cytogenet* **92**: 130–134

Bebb DG, Steele PP, Warrington PJ, Moffat JA and Glickman BW (1999) Absence of mutations in the ATM gene in forty-seven cases of sporadic breast cancer. *Br J Cancer* **80**: 1979–1981

Bishop DT and Hopper J (1997) A-T-tributable risk? *Nat Genet* **15**: 22

Boder E (1985) Ataxia-telangiectasia: an overview. *Kroc Foundation Series* **19**: 1–63

Broeks A, Urbanus JHM, Floore AN, Dahler EC, Klijn JG, Rutgers EJ, Devilee P, Russell NS, van Leeuwen FE and van't Veer L (2000) ATM heterozygous germline mutations contribute to breast cancer susceptibility. *Am J Hum Genet* **66**: 494–500

Bukholm IK, Nesland JM, Kåresen R, Jacobsen U and Børresen AL (1997) Relationship between abnormal p53 protein and failure to express p21 protein in human breast carcinomas. *J Pathol* **181**: 140–145

Chen J, Birkholtz GG, Lindblom P, Rubio C and Lindblom A (1998) The role of ataxia-telangiectasia heterozygotes in familial breast cancer. *Cancer Res* **58**: 1376–1379

Easton DF (1994) Cancer risks in A-T heterozygotes. *Int J Radiat Biol* **66**: 177–182

FitzGerald MG, Bean JM, Hegde SR, Unsal H, MacDonald DJ, Harkin DP, Finkelstein DM, Isselbacher KJ, Haber DA (1997) Heterozygous ATM mutations do not contribute to early onset of breast cancer. *Nat Genet* **15**: 307–310

Gatti RA, Tward A and Concannon P (1999) Cancer risk in ATM heterozygotes: a model of phenotypic and mechanistic differences between missense and truncating mutations. *Mol Genet Metab* **68**: 419–423

Inskip HM, Kinlen LJ, Taylor AM, Woods CG and Arlett CF (1999) Risk of breast cancer and other cancers in heterozygotes for ataxia-telangiectasia. *Br J Cancer* **79**: 1304–1307

Izatt L, Greenman J, Hodgson S, Solomon E. (1999) Identification of germline missense mutations and rare allelic variants in the ATM gene in early-onset breast cancer. *Genes Chrom Cancer* **26**: 286–294

Janin N, Andrieu N, Ossian K, Lauge A, Croquette MF, Griscelli C, Debre M, Bressac-de-Paillerets B, Aurias A and Stoppa-Lyonnet D. (1999) Breast cancer risk in ataxia telangiectasia (AT) heterozygotes: haplotype study in French AT families. *Br J Cancer* **80**: 1042–1045

Laake K, Ødegard A, Andersen TI, Bukholm IK, Kåresen R, Nesland JM, Ottestad L, Shiloh Y, Børresen-Dale AL (1997) Loss of heterozygosity at 11q231 in breast carcinomas: indication for involvement of a gene distal and close to ATM. *Genes Chrom Cancer* **18**: 175–180

Laake K, Telatar M, Geitvik GA, Hansen RØ, Heiberg A, Andresen AM, Gatti R, and Børresen-Dale AL (1998) Identical mutation in 55% of the ATM alleles in

 © 2000 Cancer Research Campaign

11 Norwegian AT families: evidence for a founder effect. *Eur J Hum Genet* **6**: 235–244

Laake K, Jansen L, Hahnemann JM, Brøndum-Nielsen K, Lönnqvist T, Kääriäinen H, Sankila R, Lähdesmäki A, Hammarström L, Yuen J, Tretli S, Heiberg A, Olsen JH, Tucker M, Kleinerman R and Børresen-Dale A-L (2000) Characterisation of *ATM* mutations in 41 Nordic families with ataxia telangiectasia. *Hum Mutat* **16**: 232–246

Larson GP, Zhang G, Ding S, Foldenauer K, Udar N, Gatti RA, Neuberg D, Lunetta KL, Ruckdeschel JC, Longmate J, Flanagan S and Krontiris TG (1998) An allelic variant at the *ATM* locus is implicated in breast cancer susceptibility. *Genet Test* **1**: 165–169

Nichols KE, Levitz S, Shannon KE, Wahrer DC, Bell DW, Chang G, Hedge S, Neuberg D, Shafman T, Tarbell NJ, Mauch P, Ishioka C, Haber DA and Diller L (1999) Heterozygous germline ATM mutations do not contribute to radiation-associated malignancies after Hodgkin's disease. *J Clin Oncol* **17**: 1259

Olsen JH, Hahnemann JM, Børresen-Dale A-L, Brøndum-Nielsen K, Kleinermann R, Kääriäinen H, Hammarstrom L, Lähdesmäki A, Lönnqvist T, Sankila R, Seersholm N, Tretli S, Yuen J, Boice J Tucker M (2000). Cancer in patients with ataxia-telangiectasia and their relatives – a collaborative study of the Nordic countries. *JNCl*: in press

Oppitz U, Bernthaler U, Schindler D, Sobeck A, Hoehn H, Platzer M, Rosenthal A, Flentje M (1999) Sequence analysis of the ATM gene in 20 patients with RTOG grade 3 or 4 acute and/or late tissue radiation side effects. *Int J Radiat Oncol Biol Phys* **44**: 981–988

Nichols KE, Levitz S, shannon KE, Wahrer DC, Bell DW, Chang G, Hegde S, Neuberg D, Shafman T, Tarbell NJ, Mauch P, Ishioka C, Haber DA, Diller L (1999) Heterozygous germline ATM mutation do not contribute to radiation-associated malignancies after Hodgkin's disease. *J Clin Oncol* **17**: 1259

Regueiro JR, Porras O, Lavin M, Gatti RA (2000) Ataxia-telangiectasia – a primary immunodeficiency revisited. *Immunol Allerg Clin North Am* **20**: 177–206

Savitsky K, Bar-Shira A, Gilad S, Rotman G, Ziv Y, Vanagaite L, Tagle DA, Smith S, Uziel T, Sfez S, Ashkenazi M, Pecker I, Frydman M, Harnik R, Patanjali SR, Simmons A, Clines GA, Sartiel A, Gatti RA, Chessa L, Sanal O, Lavin MF, Jaspers NGJ, Taylor AMR, Arlett CF, Miki T, Weissman TS, Lovett M, Collins FS and Shiloh Y. (1995) A single ataxia telangiectasia gene with a product similar to Pl-3. kinase. *Science* **268**: 1749–1753

Sedgwick RP and Boder E (1991) Ataxia-telangiectasia. In: Vinken PJ, Bruyn GW, and Klawans HL (eds) *Handbook of Clinical Neurology*, pp 347–423. Elsvier Science: New York

Shayeghi M, Seal S, Regan J, Collins N, Barfoot R, Rahman N, Ashton A, Moohan M, Wooster R, Owen R, Bliss JM, Stratton MR, Yarnold J (1998) Heterozygosity for mutations in the ataxia telangiectasia gene is not a major cause of radiotherapy complications in breast cancer patients. *Br J Cancer* **78**: 922–927

Swift M, Morrell D, Massey RB and Chase CL (1991) Incidence of cancer in 161 families affected by ataxia-telangiectasia. *N Engl J Med* **325**: 1831–1836

Taylor AM, McConville CM, Rotman G, Shiloh Y and Byrd PJ (1994) A haplotype common to intermediate radiosensitivity variants of ataxia-telangiectasia in the UK. *Int J Radiat Biol* **66**: 35–41

Vorechovsky I, Luo L, Lindblom A, Negrini M, Webster AD, Croce CM, Hammarström L (1996*a*) ATM mutations in cancer families. *Cancer Res* **56**: 4130–4134

Vorechovsky I, Rasio D, Luo L, Monaco C, Hammarstrom L, Webster AD, Zaloudik J, Barbanti-Brodani G, James M, Russo G (1996*b*) The ATM gene and susceptibility to breast cancer: analysis of 38 breast tumors reveals no evidence for mutation. *Cancer Res* **56**: 2726–2732

*© 2000 Cancer Research Campaign* 
